# Continuous remote monitoring of COPD patients—justification and explanation of the requirements and a survey of the available technologies

**DOI:** 10.1007/s11517-018-1798-z

**Published:** 2018-03-05

**Authors:** Ivan Tomasic, Nikica Tomasic, Roman Trobec, Miroslav Krpan, Tomislav Kelava

**Affiliations:** 1 Division of Intelligent Future Technologies, Mälardalen University, Högskoleplan 1, 72123 Västerås, Sweden; 2 Department of Clinical Sciences, Lund University, Lund, Sweden; 3 Department of Neonatology, Karolinska University Hospital, Stockholm, Sweden; 4 Department of Communication Systems, Jozef Stefan Institute, Ljubljana, Slovenia; 5 Department of Cardiology, University Hospital Centre, Zagreb, Croatia; 6 Department of Physiology, School of Medicine, University of Zagreb, Zagreb, Croatia

**Keywords:** Remote patient monitoring, Telehealthcare, Telemedicine, Telehealth, eHealth, Chronic obstructive pulmonary disease, COPD, Patch ECG, Transcutaneous measurement, Decision support in healthcare

## Abstract

Remote patient monitoring should reduce mortality rates, improve care, and reduce costs. We present an overview of the available technologies for the remote monitoring of chronic obstructive pulmonary disease (COPD) patients, together with the most important medical information regarding COPD in a language that is adapted for engineers. Our aim is to bridge the gap between the technical and medical worlds and to facilitate and motivate future research in the field. We also present a justification, motivation, and explanation of how to monitor the most important parameters for COPD patients, together with pointers for the challenges that remain. Additionally, we propose and justify the importance of electrocardiograms (ECGs) and the arterial carbon dioxide partial pressure (PaCO_2_) as two crucial physiological parameters that have not been used so far to any great extent in the monitoring of COPD patients. We cover four possibilities for the remote monitoring of COPD patients: continuous monitoring during normal daily activities for the prediction and early detection of exacerbations and life-threatening events, monitoring during the home treatment of mild exacerbations, monitoring oxygen therapy applications, and monitoring exercise. We also present and discuss the current approaches to decision support at remote locations and list the normal and pathological values/ranges for all the relevant physiological parameters. The paper concludes with our insights into the future developments and remaining challenges for improvements to continuous remote monitoring systems.

**Graphical abstract:**
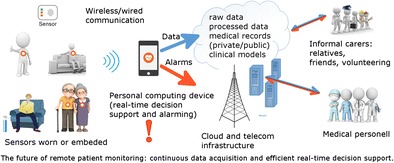
ᅟ

ᅟ

## Introduction

According to the World Health Organization (WHO) [1], chronic obstructive pulmonary disease (COPD) is currently the fourth, and will soon become the third, most frequent cause of death worldwide. It is also a disabling disease and therefore associated with high costs for treating and managing patients.

At the same time, it is well known that remote patient monitoring can significantly reduce healthcare costs [2]. Such remote monitoring is very applicable to COPD patients, and there is evidence that it reduces costs [3] by at least 14% [4]. However, there are still no widely accepted remote monitoring services involving recently developed sensors that use available technologies efficiently. Currently, COPD patients’ physiological parameters are not continuously monitored outside hospitals, except for research purposes. Symptoms are typically reported by the patients themselves using paper-based diaries.

COPD patients are usually outpatients, except in cases of exacerbation (sudden worsening of their health status, which happens on average one to four times per year), for which they might be hospitalized. More than 70% of COPD-related healthcare costs are consequences of emergency and hospital stays for the treatment of exacerbations [5]. On the other hand, remote monitoring can reduce the frequency and severity of COPD exacerbation symptoms [6], and consequently reduce the costs. Moreover, there are studies that investigate the suitability of home care for severe, uncomplicated exacerbations [7].

Even though economic aspects are important, the primary goal of remote monitoring is an improvement in the level of care. Based on 10 trials, McLean et al. showed in a Cochrane systematic review that telehealthcare can increase the quality of life for COPD patients, estimated by the St. George’s Respiratory Questionnaire (SGRQ) [8], and reduce the number of exacerbations and emergency hospitalizations [9]. The authors defined the term telehealthcare as follows: “healthcare at a distance, involving the communication of data from the patient to the health caregiver, usually a doctor or nurse, who then processes the information and responds with feedback about the management of the illness.”

The terms telemonitoring and telemedicine are probably the most often used synonyms for telehealthcare, even though the term telemedicine most often refers only to communications between healthcare providers [10]. The two terms are used by Paré et al. [11, 12] and Bashshur et al. [13], respectively, in their reviews that demonstrate the usefulness of remote monitoring in chronic diseases in general. A recent review by Lundell et al. showed evidence that “telehealthcare may lead to improvements in physical activity level” for COPD patients [14].

A review by Pedone and Lelli [15], including an investigation involving 256 patients in the UK [16] that employed SGRQ and oxygen saturation monitoring, showed the positive but not significant effect of telehealthcare on hospital admissions and emergency room (ER) visits. In agreement, McDowell et al. [17] showed that home-based healthcare with telemonitoring improves the SGRQ score, but did not provide any significant improvement in ER visits, hospital admissions, and exacerbations, and was not even cost effective.

In conclusion, the evidence for telehealthcare’s effectiveness is mixed and not conclusive, but the studies in general show an improvement or at least an equivalence with the standard care, without differences in mortality rates (except for the review by Polisena et al. [18], which showed higher mortality rates in the “telephone-support” group).

These studies of telehealthcare focus mostly on the use of communication technologies, like telephone and internet, to communicate with healthcare professionals from home, instead of the usual face-to-face care, whereas the physiological data and symptom self-reporting are transmitted and analyzed on a daily basis. As such, telehealthcare is a subset of “remote monitoring” as we define it, which also encompasses automatic continuous physiological data transmission and processing, decision support, the prediction of deteriorations, and alarming. Therefore, we can expect that remote monitoring, as it develops over time and involves more measured parameters and more sophisticated decision support algorithms, will be more efficient than telehealthcare is today.

In recent years, the developments in remote monitoring solutions have been intensive. Consequently, there are a number of different devices that can be used for monitoring the physiological, behavioral, and environmental parameters for COPD patients [19]. However, there are still various challenges when it comes to obtaining all the significant sensory inputs, and about how to merge and fuse the data coming from different sources and sensors, for the purposes of providing the input to a decision support system that should provide efficient early detection, prediction, and alarms for medical personnel or other healthcare providers. The most important task of any alarm system is to detect common life-threatening events in a timely manner, in order that an effective treatment is possible.

We see four possibilities with respect to the remote monitoring of COPD patients: (1) continuously monitoring during normal daily activities for the early detection of exacerbations and life-threatening events, (2) during the home treatment of mild exacerbations, (3) for monitoring oxygen therapy, and (4) for monitoring exercise.

With respect to the technology used, most of the previous efforts related to enabling the remote monitoring of COPD patients focused on using the self-reporting of symptoms, pulse oximetry, and spirometry [20, 21]. Medical justifications for the choices in sensory inputs will be explained in the continuation, together with the current state of the art. Pulse oximeters, however, cannot detect changes in the arterial carbon dioxide (CO_2_) partial pressure (PaCO_2_), which we argue is one of the most significant parameters for COPD patients. The PaCO_2_ is currently obtained invasively by taking blood samples at discrete time instants. To motivate an improvement, this paper justifies PaCO_2_ monitoring for COPD patients continuously and noninvasively by transcutaneous CO_2_ measurements (PtcCO_2_). We also discuss and justify the role of cardiac electrical activity, i.e., electrocardiogram (ECG) monitoring. PtcCO_2_ and ECG have not been used significantly so far, even though they are two crucial physiological parameters for the remote monitoring of COPD patients. There are currently specific scientific and technological challenges for the continuous remote monitoring of the PtcCO_2_ and ECG, which will also be discussed and explained in the continuation.

## Chronic obstructive pulmonary disease—a short description for engineers

COPD is a chronic and progressive respiratory disease for which there is no cure for the cause of the disease, but there are a number of therapeutic approaches for relieving the symptoms and slowing the progression of the disease. It presents as a combination of signs and symptoms, of which breathlessness, excessive sputum production, reduced exercise tolerance, and a chronic cough are the most common. The main causes of COPD are tobacco smoke (the prevalent cause), respiratory infections, air pollution, dust, and chemicals in poorly ventilated areas.

The pathology of COPD affects the large airways, the small (peripheral) bronchioles, and the lung tissue itself. The normal inflammatory response in the aforementioned clinical conditions is amplified in people prone to COPD development. The pathophysiologic mechanisms are not clear and are most likely diverse. Increased numbers of activated polymorphonuclear leukocytes and macrophages release elastases, the enzymes responsible for degradation, in a manner that cannot be counteracted effectively, resulting in destruction of the lung [22]. As a consequence of the destroyed alveolar walls, the total respiratory surface area responsible for gas exchange is reduced. In addition, the increased airflow resistance in the large and small airways causes difficulties with breathing, air trapping in the lung, and hyperinflation of the lung. The air trapped within the destroyed lung forms large air pockets that are poorly ventilated (areas with perfusion but no ventilation). The impaired regional ventilation causes a ventilation-perfusion (V/Q) mismatch.

Easily noticeable physiological consequences are difficulties in exhaling and breathlessness. In patients with severe COPD, poor sleep quality is often reported [23]. Exacerbation episodes are important and are the triggers for bacterial or viral infections, necessitating antibiotic treatment.

In patients that smoke, the cessation of tobacco use is mandatory. In fact, this is the only intervention that definitely slows the development of COPD. When the disease is diagnosed, vaccination against influenza and pneumococcal pneumonia is recommended, since influenza is the most important cause of excess mortality. As an anti-inflammatory treatment for patients with frequent exacerbations, inhaled or oral corticosteroids are usually prescribed for short intervals. A possible additional therapy is pulmonary rehabilitation (a comprehensive program that combines exercise training, smoking cessation, nutrition counseling, and education) followed by a home exercise program and/or by repeated pulmonary rehabilitations.

Another common therapeutic intervention is home oxygen admission, each day for at least 15 h, to ensure the maximum benefit [24] (also during sleep), or at least during planned exercise, where there is evidence that it improves exercise performance [25] and endurance [26]. This long-term oxygen therapy (LTOT) improves the survival rates for COPD patients with low arterial oxygen partial pressure (PaO_2_) (less than 7.3 kPa (55 mmHg)) [27]. LTOT can improve outcomes other than mortality, including quality of life, cardiovascular morbidity, depression, cognitive function, exercise capacity, and frequency of hospitalization [28]. Oxygen in LTOT is delivered to the nose with nasal cannulas (prongs). The drawbacks of LTOT are that it additionally reduces the mobility of patients, can increase levels of CO_2_ in the blood stream, and entails a risk of burns and starting fire for patients who continue to smoke, since oxygen supports burning [29].

To prevent death from a lack of oxygen, oxygen is also commonly administrated in acute COPD exacerbations, most often by means of non-invasive ventilation (NIV), which in contrast to LTOT provides a positive pressure during breathing, and is therefore delivered by a mask over nose and mouth [30]. Another indication for NIV (or even invasive ventilation) is the exhaustion that can be caused by increased work of breathing.

### Oxygen levels and carbon dioxide retention in patients with COPD

CO_2_ and O_2_ are the gases exchanged between the blood and the inhaled air during respiration, with CO_2_ diffusing approximately 20 times faster than O_2_. The exchange of gases is compromised in COPD because of the destroyed airways and lung tissue, which appears as the ventilation/perfusion ratio (V/Q) mismatch. As a consequence of respiratory failure, either only the PaO_2_ is decreased, or both the PaO_2_ and PaCO_2_ are abnormal (low PaO_2_—hypoxemia, high PaCO_2_—hypercapnia).

The gold standard for obtained blood-gas partial pressures and saturations is the arterial blood gas test (ABG), which involves taking an arterial blood sample and is therefore invasive. Oxygen levels are routinely measured noninvasively by pulse oximetry, which provides continuous peripheral O_2_ saturation (SpO_2_). The SpO_2_ is usually approximately equal to the arterial oxygen saturation (SaO_2_) that is obtained by ABG (the typical difference is < 2% [31]). The SaO_2_ and PaO_2_ are directly related through the oxygen–hemoglobin dissociation curve (ODC), which is not fixed and can vary even for the same person, not only depending on the temperature, PaCO_2_, and pH but also depending on some pathological conditions [31]. On the other hand, PaCO_2_ is routinely estimated noninvasively from the exhaled breath (end-tidal CO_2_ pressure (PetCO_2_)).

The PetCO_2_, however, is not a reliable estimate of the PaCO_2_ for COPD patients because of physiological reasons [32, 33], and because the gas needs to be sampled directly at the patient’s airways [34] which is practically hard to accomplish. Fortunately, novel research shows that it is possible to reliably estimate the PaCO_2_ and even PaO_2_ from transcutaneous monitoring [33, 35–46] (PtcCO_2_ and PtcO_2_, respectively), which is not affected by the COPD specificities and is therefore reliable for COPD patients. In fact, PtcCO_2_ has recently become the preferred method for estimating PaCO_2_ in remote settings [32, 33].

Oxygen is commonly administered to patients during LTOT, but more importantly in acute exacerbations, which saves lives by preventing severe hypoxemia. Oxygen therapy can, however, increase the PaCO_2_, as explained in the next paragraph. In fact, about a quarter of COPD patients with acute exacerbations are at risk of hypercapnia if they are given a high dose of oxygen [47].

**Fig. 1:**
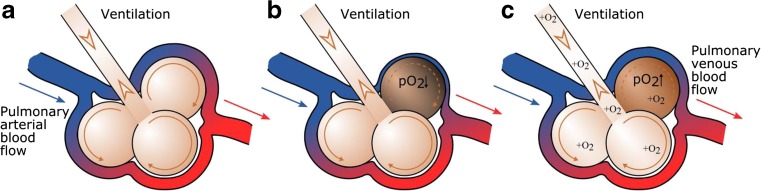
Hypoxic pulmonary vasoconstriction and its inhibition by oxygen therapy. **a** Normal alveolar ventilation and perfusion. **b** Reduced ventilation in the darker alveolus (thus, the local pO_2_ also drops) causes reduced perfusion. **c** Oxygen therapy increases pO_2_ in all the alveoli, including the dark one, and inhibits hypoxic pulmonary vasoconstriction
- Another mechanism for oxygen-induced hypercapnia is the Haldane effect, which refers to the increased capacity of deoxygenated hemoglobin to bind and carry CO_2_, compared to the oxygenated form. Thus, when there is an abundance of O_2_, CO_2_ is released into the blood stream, causing the PaCO_2_ to rise [48].
- People with healthy lungs have breathing regulation dependent mostly on changes in blood CO_2_ levels. Since some patients with COPD have high levels of CO_2_ for long periods of time, the brain’s regulatory breathing center can, over time, become less sensitive to CO_2_ levels, and more dependent on O_2_ levels, which causes these patients to rely more on a low arterial O_2_ level to stimulate their breathing. This means that oxygen therapy can reduce the stimulus to breathe. As a consequence of this reduced breathing, CO_2_ removal from the lungs is decreased. This “hypoxic drive” theorem was traditionally widely accepted, but it was recently challenged at least for the acute situation, as having only a time-limited effect on ventilation that cannot explain the total increase in PaCO_2_ [48].

It might seem absurd at first glance that oxygen therapy can cause hypercapnia, but there are several mechanisms responsible for this effect [47]. Here, we mention the most influential:

- Oxygen-induced deterioration of V/Q matching (see Fig. 1). As was already mentioned before, COPD is characterized by V/Q mismatching. There is, however, a physiological mechanism that is correcting the V/Q ratio: for alveoli with reduced ventilation (thus reduced alveolar pO_2_—partial pressure of oxygen), pulmonary capillaries supporting these alveoli will constrict, reducing perfusion for the alveoli with reduced ventilation, and consequently improving the V/Q match. In other words, the delivery of blood is reduced to poorly ventilated parts of the lung, causing more blood to go to areas where the gases can be more efficiently exchanged. This mechanism is called hypoxic pulmonary vasoconstriction. The most influential factor for hypoxic pulmonary vasoconstriction is alveolar pO_2_. During the oxygen therapy, the pO_2_, even in alveoli with low ventilation, will increase, inhibiting hypoxic pulmonary vasoconstriction. As a result, alveoli with relatively impaired ventilation will be well perfused, leading to an increase in V/Q mismatch [48]. Put more intuitively, a perfusion increase in the lung areas that are purely ventilated will decrease the efficiency of the gas exchange and cause an increase in PaCO_2_.

Hypercapnia can cause various symptoms, ranging from mild headaches, lethargy, and confusion, to severe ones with a hypnotic effect and acidosis with subsequent organ dysfunction, which can lead to coma and death [47, 49]. It also decreases diaphragm contractility and favors muscle fatigue [50]. Clinical signs of hypercapnia are as follows [47]: vasodilation producing flushing and warm peripheries with dilated blood vessels (including retinal veins), a bounding pulse (a pulse that feels full and spring-like on palpation as a result of an increased thrust of cardiac contraction or an increased volume of circulating blood within the elastic structures of the vascular system), drowsiness, flapping tremor, confusion, and coma.

To decrease the risk of hypercapnia induced by oxygen therapy in acute situations, current clinical guidelines recommend that all patients with COPD receive oxygen therapy targeted at 88–92% SpO_2_ until hypercapnia is excluded by an ABG analysis within 1 h of the treatment being started [24, 47]. For some patients, even very small amounts of supplemental oxygen are sufficient to worsen hypercapnia, so they might need an even lower target saturation range [47]. On the other hand, if the PaCO_2_ is normal, oxygen therapy can target the usual saturation range of 94–98% and so be more efficient [47].

For the purpose of keeping the SaO_2_ within a desired range, pulse oximeters are used in a closed loop to correct for the administrated oxygen levels. There is clear evidence that this titrated, and therefore closely regulated, acute oxygen therapy reduces the mortality and hypercapnia, compared to non-titrated oxygen therapies [24, 51].

LTOT is also initially titrated for specific needs of each patient but not during the therapy. The goal of the initial titration is to reach SpO_2_ > 90% (resting PaO_2_ > 8 kPa (60 mmHg)) without a significant rise in CO_2_ [52]. This is done by the ABG analysis, but transcutaneous measurements can also be used [52].

Although it is widely accepted that oxygen-induced hypercapnia can develop during COPD exacerbations, few studies have reported on PaCO_2_ changes during LTOT. Cooper et al. reported a mean PaO_2_ increase of 3 kPa, and a mean PaCO_2_ increase of 0.39 kPa, while patients were breathing oxygen (30% concentration) [53]. Török et al. showed increase in PaCO_2_ during incremental increase of oxygen flow in the initial adjustment of LTOT but the “rise in CO_2_ was not considered to be high enough to lead to a lower prescription of oxygen” [54]. During sleep, however, physiological changes occur that cause the SpO_2_ to drop for the patients with COPD (nocturnal oxygen desaturation (NOD)). Since hypoxemia has effects on the cardiovascular system, NOD could possibly also be a reason why COPD patients die more during the night [23]. To counteract NOD, there is a recommendation, inconsistently applied around the world [55], to increase oxygen flow by 1 l/min during sleep and during exercise (to counteract the increased need for oxygen during exercise) [23]. At least one study has reported a significant increase in PaCO_2_ due to this increase in oxygen flow [56]. Therefore, at least during sleep, patients could benefit from titrated LTOT, much like for acute oxygen therapy, but the patients should also be monitored for hypercapnia [23].

Even though NIV at home is currently not explicitly recommended for COPD patients [30, 57], it is used instead of LTOT for specific patients especially in some geographic areas [58]. In contrast to LTOT, transcutaneous CO_2_ measurement was studied for NIV at home as an alternative to ABGs for adjusting NIV settings and was found appropriate [33, 35, 41, 59].

### Relation between COPD and cardiac problems

If low oxygen levels are present for longer periods of time (chronic hypoxemia), they can result in hypoxic pulmonary vasoconstriction (Fig. 1). On the other hand, the destruction of lung tissue leads to a breakdown of the pulmonary capillaries, and hence a reduction of the pulmonary vascular system. These two mechanisms cause an increased resistance of the pulmonary vascular system to the blood stream. The increased resistance causes an increase in the pulmonary artery pressure and makes it harder for the heart to pump blood to the lungs. If this condition continues, it can cause the heart muscle to grow in size (right ventricular hypertrophy) and eventually lead to failure of the right side of the heart [60].

It takes time to develop right-side heart failure, but the heart remodeled with hypertrophy, and receiving hypoxemic blood for its own nutrition, is very prone to arrhythmias and sudden cardiac events. On the other hand, hypercapnia decreases myocardial contraction [61] and also predisposes to arrhythmias [62]. Furthermore, COPD occurs frequently with coronary artery disease [63]. For all these reasons, a substantial proportion of the deaths in patients with COPD is the result of cardiovascular complications [63–65].

In general, cardiovascular diseases are the most frequent comorbidities with COPD and include the following entities: coronary artery disease, heart failure (about 30% of patients with stable COPD show some degree of heart failure), arrhythmias, and hypertension (one of the most frequent comorbidities in COPD) [63, 66].

### Physical activity in COPD

Regular physical activity (PA) is recommended for all patients with stable COPD [66]. Walking is generally accepted, but also stair-climbing, treadmill, or cycling exercises are beneficial. Pulmonary rehabilitation (supervised exercise) or a home exercise program can improve the lung’s functional status.

PA in COPD patients decreases the risk of hospitalization [67], reduces the decline in lung function [67], but can also improve the general health status and decrease both disability and mortality [68, 69].

PA is drastically reduced during and after hospitalization caused by exacerbation, but even patients with milder exacerbations, which do not require hospitalization, tend to stay indoors during the exacerbation period, which lowers PA levels [67]. It might take a number of weeks for patients to recover from exacerbation, during which time they lose muscle mass as a result of reduced activity.

Patients whose arterial PaO_2_ are borderline at rest may develop worsening hypoxemia during exercise, but even patients without hypoxemia may improve exercise capacity with supplementary oxygen [26]. During exercise, a substantial CO_2_ retention (PaCO_2_ increase of more than 4 mmHg) may also occur frequently in patients with COPD, and can even result in exercise-induced hypercapnia (an elevation of PaCO_2_ levels greater than 45 mmHg (6.0 kPa). Less frequent is a significant reduction of PaCO_2_ on exertion which can go even to hypocapnic levels [70].

### COPD exacerbations

“An exacerbation of COPD is an acute event characterized by a worsening of the patient’s respiratory symptoms that is beyond normal day-to-day variations and leads to a change in medication” [66]. In plain language, patients cannot catch their breath. Such exacerbations are the major cause of COPD patients’ hospitalization, but are also associated with deteriorations of the patients’ health-related quality of life [71], contribute to a long-term decline in lung function [72], and have an independent negative impact on the patients’ prognosis [73]. Additionally, mortality increases with the frequency of severe exacerbations, particularly if these require admission to a hospital [73].

Early recognition of exacerbation symptoms and prompt treatment improves recovery rates, reduces the risk of hospitalization, and is associated with a better health-related quality of life [74]. It is therefore beneficial to detect the first signs of exacerbations or to predict them so that the patients can be appropriately treated in a timely manner and optionally to be prioritized by home healthcare professionals. Nevertheless, if the exacerbations happen despite the preventative efforts, some of the patients, depending on the exacerbation severity, could still be treated at home for uncomplicated exacerbations [7, 75], since these exacerbations would not require an intensive investigation or complex therapy. According to the Global Initiative for Chronic Obstructive Lung Disease (GOLD) strategy document [30, 66], more than 80% of exacerbations can be managed on an outpatient basis. Concordantly, the European Respiratory Society published a consensus statement stating that “most exacerbations are mild and can be treated on an outpatient basis (home care).” Severe events should be evaluated in the emergency department of a hospital, so that the patient can be admitted, if necessary. If the severity of an episode is in doubt, the assessment should take place in a hospital. In a very severe, life-threatening episode, direct admission to an intensive care unit (ICU) is indicated [76].

In the case of home care, the patients can be periodically visited by respiratory nurses who monitor treatment compliance and progress. The treatment at home can be leveraged and made more efficient and secure by using continuous remote monitoring.

## Existing devices and technologies suitable for the remote and continuous monitoring of COPD patients

The monitoring of patients is supported by research and development in sensor technology, which has resulted in various wearable and unobtrusive sensors. These sensors are capable of monitoring physical activities and measuring physiological and biochemical parameters like electrocardiogram (ECG), heart rate (HR), blood pressure, PaO_2_, and body temperature [19]. There are two possibilities for unobtrusive remote monitoring: sensors can either be worn by the monitored subject or embedded in the monitored subject’s environment. Currently, noncontact sensing is prone to a low signal-to-noise ratio and is therefore not considered in this paper.

### Transcutaneous O_2_ and CO_2_ monitoring

Various devices offer the possibility to simultaneously monitor the level of oxygenation and CO_2_, combining either SpO_2_ with PtcCO_2_ (e.g., SenTecTM, Therwil, Switzerland [37], and TCM ToscaTM, Radiometer, Neuilly Plaisance, France [77]), or transcutaneous PtcO_2_ with PtcCO_2_ (e.g., TCM Combi MTM, Radiometer, Neuilly Plaisance, France [77]).

### Cardiac electrical activity (ECG) remote monitoring

**Fig. 2:**
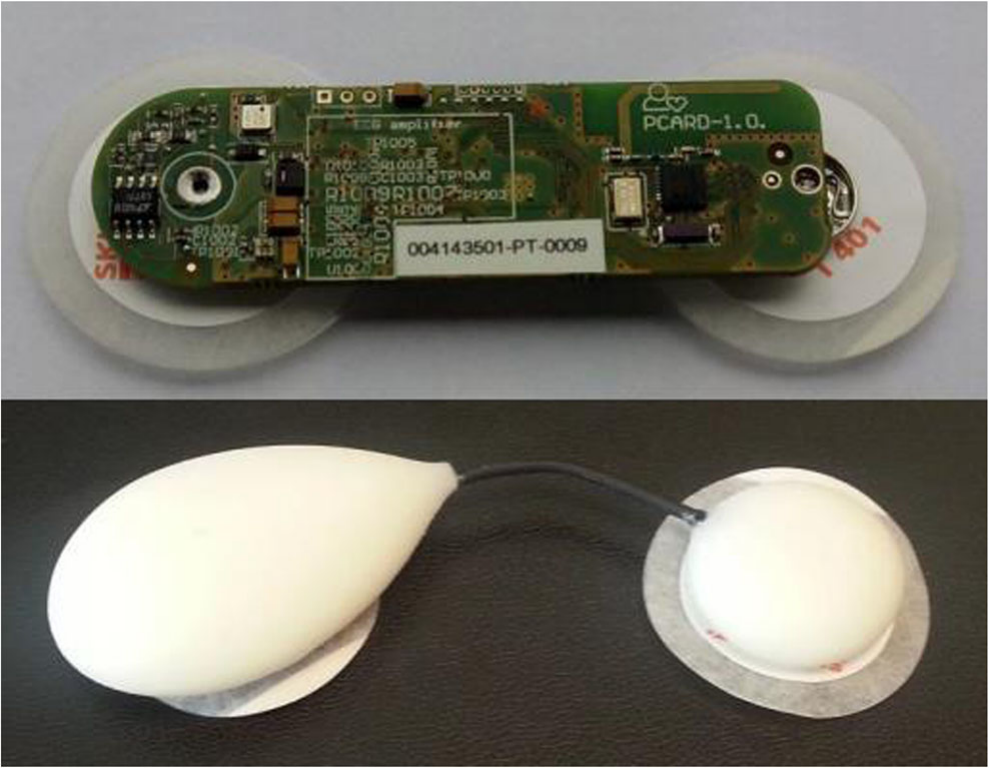
A prototype of a patch ECG monitor developed at the Jožef Stefan Institute, Ljubljana Slovenia [81, 82], with two self-adhesive disposable electrodes, a lithium coin battery, micro-processor, BT4 radio, and printed circuit board antenna (up). Savvy sensor, the final product (down)

Holter monitors are traditionally used for ECG acquisitions in remote settings, but they are being superseded by wireless bi-potential patch devices [78–80], whose development has been intensive in recent years (a representative example, a Savvy patch ECG sensor, is shown in Fig. 2). The patch ECG (PECG) sensors are already established as patient friendly, well tolerated, unobtrusive, wire free, and with recording capabilities that span from weeks to months (much longer than standard Holter monitors), which is beneficial for detecting potentially malignant arrhythmias that take a longer time to detect. They provide good adhesion to the skin, and some of them even claim to be waterproof.

The Savvy monitor has been certified as a medical device according to the directive MDD 93/42/EEC, and standards EN 60601 and EN ISO 14971, and has recently become available on the market, while the ZIO® XT Patch by iRhythm Technologies, Inc. and SEEQ™ MCT patch device by Medtronic, Inc. have already been extensively evaluated. The evaluations show evidence of an increased diagnostic lead in both adult and pediatric patients compared to Holters [79]. There are also other PECGs available on the market, and the number is expected to increase in the future [78–80].

### Respiratory rate

One of the signs of COPD exacerbation is a respiratory rate (RR) over 25/min (see Sect. 4.7). Furthermore, there is evidence that a change in RR has a strong predictive power for exacerbation [83, 84].

The respiratory effort can be captured by an impedance pneumograph, stretch sensors, and flow thermography, which captures breathing from the temperature difference between the inhaled and exhaled air. The impedance pneumogram is the most common method used in hospitals [85]. Its basic principle is to inject current into the tissue by employing two drive electrodes placed on the chest and to measure the potential difference between the two points. The potential difference is related to the impedance of the tissue, which varies with respiration.

The respiration also affects the cardiovascular physiology. Consequently, the RR can also be estimated from the ECG and pulse oximetry. Even though it is inferior to the ECG-derived RR, pulse oximetry can also provide a reliable RR [86]. Indeed, recently, the Nellcor Respiration Rate System received CE mark approval. In addition to SpO_2_, and HR monitoring, it also provides RR, and all this is obtained from a single finger sensor [87].

### Pulse oximetry

Pulse oximetry is the continuous measurement of saturation for peripheral oxygen (SpO_2_). A clip-like device is placed on a thin body part, most commonly on a finger, but also on an ear lobe, or across a foot if applied on infants. There are a number of inexpensive and easy-to-use pulse oximetry devices available on the market.

The basic principle is to emit two different wavelengths from one side of the body part using a pair of small, light-emitting diodes and measure the amount of light that passes through the body with a photodiode placed on the other side. The two wavelengths are chosen so that their absorption (the emitted minus the passed-through light) is significantly different for the oxygenized and not-oxygenized hemoglobin. The SpO_2_ is calculated from the ratio of the two measurements.

### Nutrition and weight monitoring

Both obesity and loss of body mass are commonly encountered with COPD [76]. Malnutrition is associated with respiratory muscle dysfunction and increased mortality. Therefore, nutritional intervention should occur if a large loss of body mass is detected. Nutritional interventions aimed at achieving an ideal body weight are also recommended. High-carbohydrate diets and an extremely high caloric intake should be minimized to reduce the risk of excess CO_2_ production [76]. Furthermore, obesity contributes to breathlessness in some individuals due to affected diaphragm mobility. On the other hand, weight loss is associated with a more severe impairment of the lung function [76].

### Blood pressure measurement

The justification for measuring blood pressure comes from the fact that hypertension is a frequent comorbidity in COPD [66], but can also be caused by corticosteroid therapy. “Blood pressure” normally refers to the arterial pressure in the systemic circulation. There are also measurements of pressures in the venous system and pulmonary vessels, but these require a catheter and are therefore beyond the scope of this paper.

The arterial blood pressure is noninvasively routinely measured by the auscultatory method involving an inflatable cuff wrapped around the arm, a manometer measuring the pressure in the cuff, and a stethoscope placed over the brachial artery at the elbow. There is also the oscilometric method, which is more suitable for automatic measurements in home environments [88]. The oscilometric method also uses a cuff. There are also continuous, noninvasive arterial pressure-monitoring devices that measure in real time, completely noninvasively and without cuffs. These devices are, however, still to prove their precision [89]. An overview of experimental blood pressure measurement devices is provided in [90].

There are a number of devices available on the market that can be used for the remote automatic monitoring of blood pressure (mostly oscilometric) that patients can use without assistance. For specific guidelines on how to measure blood pressure remotely at home, the reader can refer to [88].

### Spirometry

**Fig. 3:**
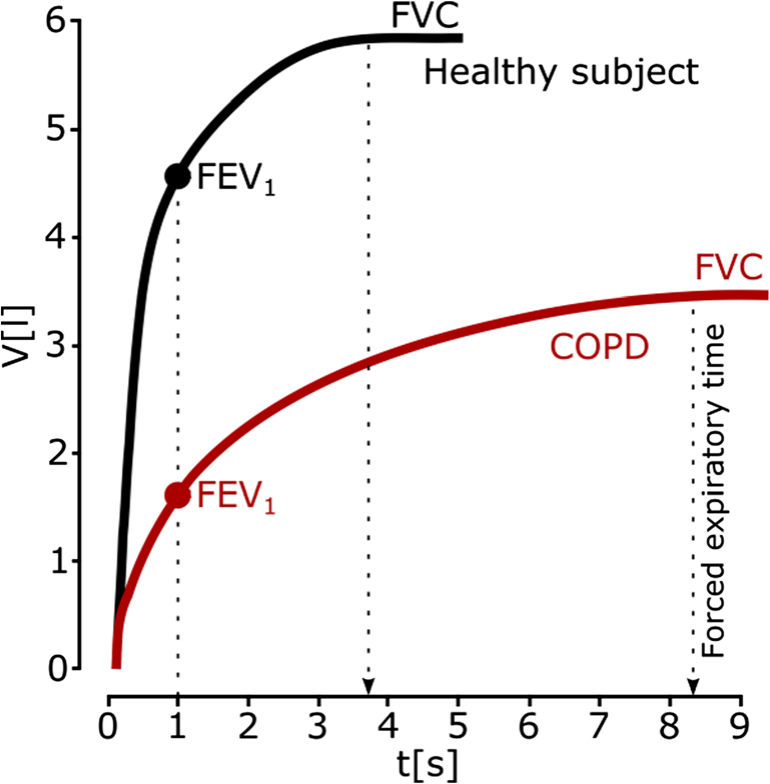
Representative spirograms (expired volume–time curves) for healthy subjects (FEV1/FEV ~ 4.6/5.8 = 0.8) and subjects with COPD (FEV1/FEV ~ 1.7/3.4 = 0.5)

Diagnostic spirometry measures the volumes of air inspired and expired by the lungs to assess a patient’s lung function for the purposes of comparison with a patient population or with previous measurements from the same patient. The presence of airflow limitation is recognized by a reduction in the ratio FEV_1_/VC or the ratio FEV_1_/FVC, where VC (vital lung capacity) is the maximum amount of air expired from the fully inflated lung, FVC (forced vital capacity) is the forced amount of air a person can exhale after the maximum inspiratory effort, and FEV_1_ (forced expiratory volume in 1 s) is the fraction of the FVC expired during the first second (see Fig. 3). The presence of a post-bronchodilator FEV_1_/FVC of less than 0.7 confirms the presence of COPD [66]. The FEV_1_/VC ratio is a relatively sensitive index of mild COPD. In moderate-to-severe cases of the disease, however, the severity of the airflow limitation is best assessed by the FEV_1_ in relation to reference values (also known as predicted values) [91].

Spirometry is used mainly in the diagnosis of COPD, as well as in the assessment of its severity, progression, and prognosis, but it is not suitable for acute states. Patients with acute severe exacerbations of COPD can be too breathless to undertake spirometry testing, but in any case, conducting spirometry in acute situations is not very informative [66].

Due to the growing number of patients, the diagnosis of COPD should take place in general practices that are equipped with spirometers. For the diagnosed patients, the spirometry should be carried out at least once per year to identify patients with a rapid decline in lung function [66]. To ensure quality, the spirometry should be carried out by nurses who have been trained in how to perform spirometry [92].

Portable handheld spirometers available on the market can be more or less accurate compared with conventional hospital-based equipment [92]. Still, the technology used in portable spirometers is constantly evolving, which means their performance is constantly improving [92]. Regardless of the device accuracy, the measurements obtained by patients on their own need to be taken with caution when supplied to a decision support system, and cannot be used as a substitute for the diagnostic spirometry [92].

Also beneficial are peak expiratory flow (PEF) meters, which are simpler and cheaper than spirometers. If we strictly apply our definition of spirometry, PEF meters are not spirometers because they measure only the maximum speed of expiration, not the volume of expired air. The PEF meters are successfully used in asthma monitoring, with high patient compliance, but they are beneficial in COPD monitoring as well. A value for PEF of less than 100 l/min is a sign of acute COPD exacerbation (see Sect. 4.7). Additionally, van den Berge et al. presented evidence that PEF is a predictive factor for exacerbation [93], whereas Inglesia et al. showed that PEF has an important predictive value for determining the risk of death in patients who required hospitalization for acute exacerbation of COPD [94].

### Thermometry

Normal body function depends on a relatively constant body temperature. A body temperature higher than 38.5 °C is one of the parameters indicating severe exacerbation [76].

Most common sites used to obtain body temperature readings with medical thermometers are the anus (rectal temperature), the mouth (oral temperature), under the arm (axillary temperature), and the ear (tympanic temperature).

Oral and rectal body temperatures, although slightly different, correlate well with the core body temperature. The most easy to apply continuous temperature measurement devices in remote settings, i.e., those that can be placed on the body surface (axillary and tympanic), do not follow the core body temperature well enough to recommend their use [95].

### Environmental sensors

Environmental sensors are present in the patients’ environments or are embedded into objects in the environments, like chairs, seats, mattresses, mirrors, toilet seats, and bathroom scales.

Sensors that measure environmental temperature and air quality can be used to assess the condition of patients’ living environments. There is evidence of a significant positive correlation between quality of life and air quality [96]. Air pollution has been shown to be a major COPD risk factor [97], but numerous other diseases can be caused by air pollution. Since most COPD patients are past or current smokers, it is quite informative to detect smoking. Smoking in combination with oxygen therapy is dangerous due to the risk of fire.

### Personal computing devices

**Fig. 4:**
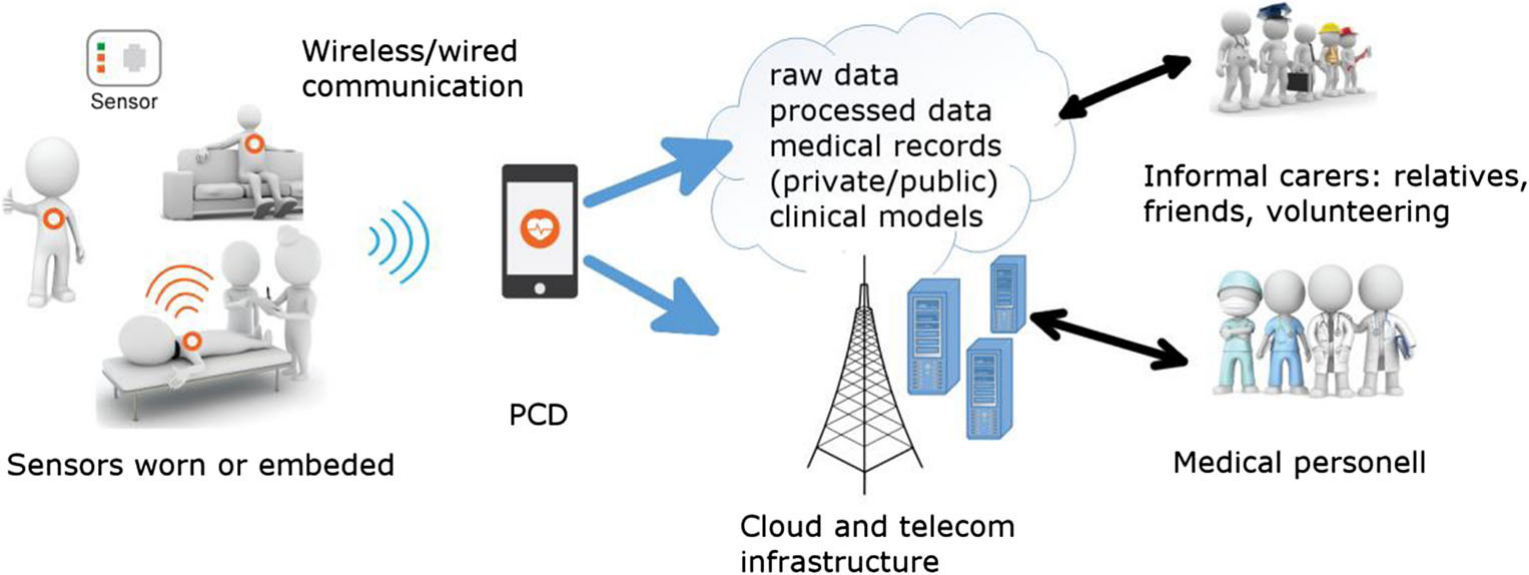
General architecture of remote monitoring systems

Data measured with physiological and environmental sensors can be preprocessed by the sensors (e.g. the R-R interval lengths can be extracted from a measured ECG). Either the results of preprocessing, or the raw signals, or both can be wirelessly transferred to the users’ personal computing devices (PCDs), such as smartphones, tablets, or smart watches. On PCDs, the data can be further processed, most importantly for detecting life-threatening events, or for some specific purposes, which in the case of an ECG can be, for example, synthesizing a 12-lead ECG [98], or extracting the breathing rate from an ECG [99]. Modern PCDs already contain a number of sensors, e.g., microphone, camera, GPS, and accelerometer. The obtained physiological signals can be integrated with the PCDs’ sensing abilities to extract relevant features that can be used as inputs for a decision support system running on the same device. From there on, the raw signals and/or the results of the processing can be forwarded to the cloud for further processing and storage [100]. The general concept of a remote monitoring system is presented in Fig. 4.

### Previous research on systems for the telemonitoring and self-management of COPD

Self-management is not universally defined, but the most general meaning of the term is the patients’ ability and skills to deal with their chronic diseases [101, 102]. In the case of self-management, patients are contacting medical professionals, whereas in telemonitoring, the communication is bidirectional and the medical personnel can initiate communication with patients or their caregivers.

A number of systems combine self-management and telemonitoring [20, 103]. Most of them focus on enabling patients to self-assess their status (see Sect. 4.5) and record pulse oximetry on their own. Some systems combine pulse oximetry with an RR sensor, blood pressure monitor, activity monitors, and less frequently some other sensors [20]. In almost all the systems, the data is transmitted on a daily basis. When a deterioration in health occurs, patients can contact the healthcare professionals or be contacted via telephone calls.

There are two available reviews covering the existing technology in COPD telemonitoring [20, 21], whereas the others, already mentioned in the introduction, focus on clinical outcomes (e.g., mortality and quality of life), reduction in healthcare service utilization, feasibility and use, and on the economic and organizational impacts of telemonitoring. Among the existing research, there is a lot of variability and a lack of consensus. For instance, different questionnaires were used as well as different threshold values for physiological parameters [21]. There is not even a consensus about what constitutes an exacerbation [104]. Efficient decision support algorithms have only been applied in a minority of studies.

The need to establish a scientifically sound methodology that is agreed on and accepted by future studies is obvious [104]. To facilitate this, in the continuation, we will identify and justify the application of the most significant technologies and sensory inputs (together with their pathological ranges) for the remote monitoring of COPD patients, review the current status of decision support systems, and present the remaining challenges.

## Scientific and technical solutions and challenges in monitoring COPD patients

### Monitoring PaCO_2_

Since oxygen therapy can increase PaCO_2_, as explained in Sect. 2.1, it is beneficial to monitor PaCO_2_ not only during acute oxygenation therapy but also possibly during LTOT (especially during sleep [23]) (see Sect. 2.1). Mortality in acute exacerbations of COPD was reported to be associated even with an increase in chronic stable levels of PaCO_2_ [51], which shows that it is also beneficial to regularly monitor stable (baseline) CO_2_ levels.

The standard for obtaining PaCO_2_ is ABG, which is an invasive procedure with risks, but also painful, time consuming, and discrete. Even though not perfectly following the PaCO_2_ obtained by ABG, transcutaneous monitoring has its advantages: it is painless, noninvasive, and continuous, with almost no sleep disturbance, and possibly self-manageable. It is therefore possible to use PtcCO_2_, along with pulse oximetry, instead of ABG, for titrating any oxygen therapy. Moreover, since PtcCO_2_ is continuous, the titration can be without delay.

PtcCO_2_ is not measured routinely in remote settings due to the lack of inexpensive PtcCO_2_ monitors. Even though it is likely that the prices of PtcCO_2_ monitors will reduce over time, as happens with all electronic devices, an open question is how to compensate for the currently high prices. One possibility is for PtcCO_2_ monitors to be used by medical personnel that periodically visit multiple COPD patients for the purpose of monitoring the baseline PaCO_2_ regularly, and/or in the minutes after the onset of oxygen therapy. This entails a need for a new software system that will keep track of each patient’s measurements and integrate them with electronic health records. Another possibility is for the same device to be shared between multiple patients for periodic use during 1 day and night. This would be enough to access how the PaCO_2_ changes during the LTOT, which is particularly important during the night, as explained in Sect. 2.1.

One current weakness associated with transcutaneous measurements is the approximate 2-min lag time for the PaCO_2_ changes to be reflected in the PtcCO_2_ [105]. An open scientific question is could the changes in PaCO_2_ be predicted to compensate for the lag?

It has been suggested that patients who are known to be at risk of hypercapnic respiratory failure should be given oxygen “alert cards” and should be instructed to show these cards to medical personnel [24, 47]. The purpose of the cards is to warn medical personnel that the patients should be given controlled rather than high-concentration oxygen therapy and to adjust the therapy based on the previous ABG results stored on the card because hypercapnic respiratory failure can occur even if the targeted SaO_2_ is below 88%. It is important to investigate how this digital alert card can be employed in a convenient form, like, for example, as a smartphone application, which could be accessible to medical personnel even if the patient is not capable of presenting the alert card on his/her own.

### ECG, ECG-derived RR, and HR

Reports show that the primary cause of death for COPD patients is cardiac failure [64, 65]. Myocardial infarction is the co-morbidity with the greatest potential for treatment and prevention to improve the prognosis of COPD patients [64]. In general, cardiovascular diseases are the most frequent and important diseases coexisting with COPD (see Sect. 2.2.) It is therefore of the greatest importance to continuously monitor cardiac electrical activity for patients at risk, and issue alarms when dangerous event are detected.

Recent developments in ECG technology have resulted in PECGs that possess convenient features for remote monitoring (see Sect. 3.2). They are small and wireless. Patients can place them on their own, freely sleep with them, and even take a shower.

They are most often single lead devices. One lead is enough to provide HR [106] which can be used to detect arrhythmias and cardiac arrest. Some arrhythmias need to be terminated instantaneously (see Sect. 5.4); therefore, it is crucial in that situation for a system of remote monitoring to alert people in the vicinity of the patient and direct them to the nearest defibrillator (which are becoming more and more present in the environment). PECGs can also be used to obtain specific indicators, like heart rate corrected QT interval (see Sect. 5.4), relevant for COPD patients.

It has also been shown that reliable RR can be estimated from ECGs produced by them [99], which eliminates the need for separate respiratory sensors. This is of additional significance for COPD patients, since RR is one of the major parameters for detecting COPD exacerbations (see Sect. 4.7).

**Fig. 5:**
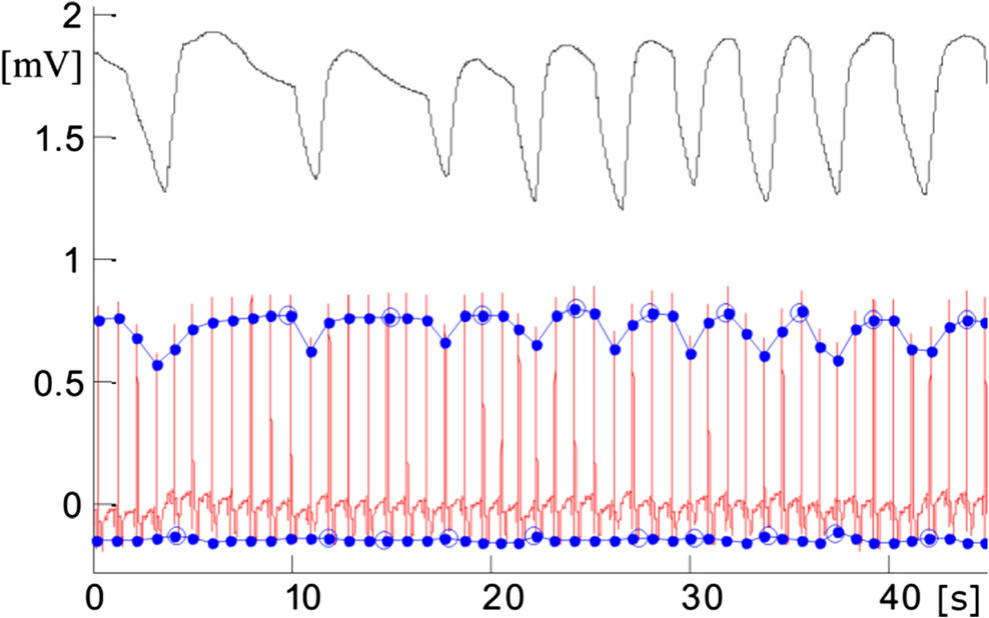
Interval of an ECG (red) measured with a patch ECG monitor, and respiration intervals (black) measured by a thermistor near the front of the nose. The amplitude of the measured ECG signal varies with respiration. All nine respiration intervals are identified (blue circles). The presented results come from a study reported in [99]

Figure 5 shows how RR can be extracted from amplitude-modulated ECG signal obtained using a PECG. For details of the algorithm used, see [99]. Respiration modulates the ECG also in terms of HR (respiratory sinus arrhythmia (RSA)). Even though it has been shown that extraction from HR provides a good approximation of the mean RR if the time series is longer than 1 min for young supine subjects, it is significantly less accurate for elderly people [107]. There are also other approaches for extracting the RR from ECG. For a recent evaluation of four different methods, i.e., filtering, R and RS amplitude, and QRS areas, please refer to [108].

Since they are still not a part of standard clinical care (and consequently not shared among patients) and are not covered by health insurance, the disadvantage of the PECGs is that they have high cumulative consumer costs. Moreover, the customers can be dependent on the device company for raw and aggregated data retrial.

The crucial current deficiency of PECG sensors is the data-processing time, which can be after the recording (ZIO XT) or after the transmission to the company’s data networks (SEEQ), where the processing time depends on the processing abilities of the company’s data center. The Savvy sensor, however, comes with stand-alone computer software for a basic analysis of the acquired ECG that a customer can use on his/her own. The data analysis algorithms that can run directly on the PECG sensor have just recently started to be developed, an example being the HeartSaver reported in [109].

Since acute life-threatening events are the most important, it is obvious that there is a need for further developments to enable wireless monitors that can not only record ECG to estimate the arrhythmia burden but more importantly detect life-threatening events in real time and send an alarm to the relevant stakeholders for a timely diagnosis and treatment.

Even though PECG sensors detect more reportable events than Holter monitors, largely because of the significantly longer recording times, there is evidence that Holter monitors detect more events in the same period of time [79]. This might be because Holters are multichannel devices, but also due to the differences in detection algorithms. Consequently, for the PECG sensors to become a standard diagnostic tool in clinical practice, it is essential for future studies to specify the abilities of each PECG sensor and the associated detection algorithms when detecting each type of arrhythmia and conduction system disease.

The PECG sensors can potentially be used even for a 12-lead ECG synthesis [98, 110, 111]. Their additional advantage is that they can be used to obtain specific ECG leads for specific purposes [112–114].

Because of an autonomic nervous system dysfunction, COPD patients have been shown to exhibit decreased HR variability compared to control subjects, as well as reduced levels of all the linear exponents and a decreased short-term fractal exponent of the intervals between beats [115]. The power of the HR-related features for predicting exacerbations remains to be investigated.

### Empowering patients and personalization

How can we make the system for the remote monitoring of COPD patients easy to use and able to adapt to each patient’s specific needs? A recent systematic review [20] shows that patients were “generally satisfied and found the systems useful to help them manage their disease and improve healthcare provision.” The review, however, indicates a number of usability problems that need to be overcome in future research. These include the short battery life, a confusing power supply, and the need to provide real-time feedback. Based on previous research by Botsis and Hartvigsen [116], the authors also emphasize that the security and confidentiality of the collected data should be satisfied. Interestingly, the authors also state that the lower compliance is related to the frequency and timing of the data collection and transmission, which is carried out at discrete time points during the day and requires effort from the patients. It is to be expected that the automatic collection and transmission of data will increase the patients’ compliance with the remote monitoring systems.

According to the study made by Gravil et al. [75], 80% of patients would be happy to be treated at home for uncomplicated exacerbations instead of being admitted to a hospital. In their study, however, the patients were visited by nurses who monitored adherence to the recommended treatment and offered reassurance, support, and education. The only measurements used were spirometry and SpO_2_, which were applied by the nurses. If the patients were to use a remote monitoring system on their own, we would expect the acceptance of the home treatment to be significantly lower. One of the reasons for this could be technophobia, which according to the American Telemedicine Association [117] can be reduced among users by tailoring systems to the specific needs of each user population. Furthermore, there is evidence that the promotion of easy-to-use systems and more training sessions, to make patients more familiar with the system, should improve the acceptance of these remote monitoring systems [20, 118]. Contributing to the acceptance should also be the provision of additional security that comes from the fact that the patients’ symptoms will be recognized early by the remote monitoring system and that they will be contacted and treated if any deterioration occurs.

One important goal of any COPD monitoring system should be to enable patients to better understand the disease, to familiarize themselves with symptoms and ways to control them, and to empower the patients to be more involved in their healthcare, so that they can recognize the exacerbations at an early stage.

### Monitoring and controlling physical activity, exercise, and reference test

Patients with COPD have significantly lower levels of physical activity (PA) than healthy controls [119], and even lower than people with some other chronic conditions [120]. Even though PA is recommended for COPD patients (Sect. 2.3), most patients do not follow these recommendations [67]. Remote monitoring may have a positive motivating effect on COPD patients to increase PA [121, 122] by providing counseling and feedback, but it is prudent to monitor COPD patients’ physiological parameters (PaCO_2_, PaO_2_, ECG) during PA also because of the health risks involved (see Sect. 2.3). For the remote assessment of PA, both questionnaires and motion sensors, like step counters and accelerometers, can be used, but a recent investigation showed that questionnaires provide overestimates of the true PA [123].

Questionnaires are mainly used for research purposes, especially in epidemiological studies. There have been more than 15 different questionnaires developed for COPD patients [67]. From the perspective of continuous monitoring, the questionnaires can be implemented in an application for PCDs and be used by the patients on an everyday basis (see Sect. 4.5).

Pedometers are devices that count the number of steps. From the number of steps counted, it is possible to estimate not only the distance traveled but also the energy expended. However, these estimates lack precision, especially when the walking is at slow speed, which is typical in patients with COPD [67]. Despite being imprecise, there is evidence that pedometers are motivating for patients to increase and maintain their levels of PA when used alone [124], or together with a PCD application providing individualized activity goals and allowing occasional telephone contacts with caregivers [122].

Accelerometers are devices that measure acceleration. The measurements obtained with accelerometers reflect body movement. For estimating PA levels and energy expenditure, they can be combined with pedometers and physiological sensors, e.g., HR and skin temperature, to provide valid estimates of PA levels [67, 125].

It is useful to know that levels of PA cannot be accurately predicted from resting lung-function parameters, i.e., spirometry [67].

There are also reference tests for evaluating the progression of the disease. One of them is the “6-min walk test” during which the patient walks the longest distance he/she can, while his/her blood saturation is monitored with a pulse oximeter to assess exercise-induced oxygen desaturation which has an additional prognostic values besides the 6-min walk distance [70]. There is evidence of a positive association between the 6-min walking distance and the PA [67].

### Self-assessment

A questionnaire implemented on a PCD can be provided to the patient to be filled in every day. They can include fields like how much coughing, how much sputum production, how much breathless—dyspnea, general feelings, PA assessment, and other questions that can be found in existing questionnaires.

The most comprehensive COPD-related questionnaires are the Chronic Respiratory Questionnaire (CRQ) [126] and the St. George’s Respiratory Questionnaire (SGRQ) [127] (online [8]). The SGRQ’s score has been associated with anxiety and depression, two major comorbidities in COPD.

The latest GOLD executive summary [30] considers CRQ and SGRQ as being “too complex to use in clinical practice” and promotes shorter measures, like the COPD Assessment test, as more suitable. Still, the questionnaires used in the research of the remote monitoring of COPD are diverse. Some of the researchers have even developed their own questionnaires [21].

A drawback of self-reporting is that it can be difficult for patients. There is evidence that only a minority of patients can “log discrete episodes of increased breathlessness, cough and purulent sputum” [128]. This is one of the reasons for preferring the continuous automatic monitoring of physiological parameters instead of self-reporting.

### Monitoring medication application and adherence

An appropriate medicament therapy can reduce COPD symptoms, reduce the frequency and severity of exacerbations, and improve health status and exercise capacity, but “existing medications for COPD have not been shown conclusively to modify the long-term decline in lung function” [66]. In particular, exacerbations are treated with bronchodilators, systemic corticosteroids, and antibiotics, and there are new drugs developed all the time [129]. The corticosteroids can cause hypertension, which calls for blood pressure measurements and ECG monitoring during corticosteroid therapy. Stopping regular medication, such as diuretics and/or bronchodilators, on the patient’s own initiative might increase symptoms [7].

It has been suggested for more than 10 years now that remote monitoring should be used as the “gold standard” for medication adherence measurements [130], but how to effectively monitor medication compliance and how to motivate patients to take their medicaments are challenges that still remain. PCD applications that provide reminders and feedback to patients could be useful as motivators. As for monitoring compliance, intelligent packaging for medicaments, equipped with electronics for collecting and transmitting the data about usage, can provide controlled access to medicaments in terms of keeping track of how many medicaments have been used and at which times. This enables monitoring of the adherence to the therapy, the evaluation of patterns of medicament use, and monitoring the dose-response relationship [130]. Obviously, smart packaging can be tricked into tracking adherence since it is not able to detect whether the pills are actually swallowed.

Perhaps the most impressive recent development in monitoring medication adherence, which cannot be easily tricked, is the Proteus Digital Health ingestible sensor that measures medication ingestion and adherence patterns in real time [131]. It is a system of a 1-mm digestible chip and a patch that picks up the signal from the chip and can also capture the HR [132]. Another example of recent developments that are particularly significant for COPD and asthma patients is AstraZeneca’s inhaler device called Turbuhaler, which has recently been accompanied by a monitoring device to monitor the actuations of the inhaler [133].

A related problem is how to tailor medications to individual needs. According to the GOLD: “Each pharmacological treatment regime needs to be adapted to the patient (i.e., individualized), guided by the severity of the symptoms, the risk of exacerbations, comorbidities, drug availability, and the patient’s response” [66]. This can be at least partially achieved by using remote monitoring, for the purpose of titrating/adjusting the treatment, or providing patients with personalized advice. For example, in stable COPD, an increase in FEV_1_ following a therapeutic trial of corticosteroids for several days is often taken as an indication of regular use for these drugs [76].

### Detection and classification of exacerbations

Signs are objective, whereas symptoms are subjective, evidence of a health problem.

The symptoms of severe COPD exacerbations that require hospitalization are [76]

1. Change in cough frequency.
2. Change in sputum production and appearance.
3. Increase in dyspnea at rest.

The signs of severe COPD exacerbations that require hospitalization are [76]

4. Inability to speak one full sentence.
5. Temperature > 38.5 °C.
6. Ankle oedema.
7. Respiratory rate > 25/min.
8. HR > 110/min.
9. PaO_2_ < 8 kPa [75].
10. Worsening cyanosis.
11. Use of accessory muscles.
12. Loss of alertness.
13. PEF < 100 l/min.

All of these parameters are measurable at home. Parameter 12 is significant on its own, whereas parameters 3, 7, 8, 10, and 11 are significant as a group [76]. It is important to note that the exact thresholds in the previous list are not universally accepted; studies in remote monitoring employ diverse exacerbation criteria [21], which might be one of the reasons for the inadequate performance of decision support algorithms (discussed in the next section).

The criteria for severe COPD exacerbations based on parameters that are normally measured in hospitals but can now also be measured in the home environment with portable spirometers, transcutaneous measurements, and portable ECG devices are [76] FEV_1_ < 1 l, PaO_2_ < 8 kPa (60 mmHg), SaO_2_ < 90%, PaCO_2_ ≥ 6.0 kPa (45 mmHg), and ECG abnormalities. Additional measurements for severe acute exacerbation, which are currently not measurable at home, are chest radiograph, white blood cell count ≥ 12,000, sputum stain/culture, biochemistry (electrolytes, urea, glucose, etc.). The life-threatening events are respiratory or cardiac arrest, confusion or coma, PaO_2_ < 6.7 kPa (50 mmHg), PaCO_2_ ≥ 9.3 kPa (70 mmHg), pH < 7.3.

COPD exacerbations can often be prevented [66]. It is therefore desirable to predict or at least early detect signs and symptoms of exacerbations. This can be done automatically by using decision support systems featuring the classification of patient states.

### Decision support (exacerbation prediction and detection algorithms)

**Fig. 6:**
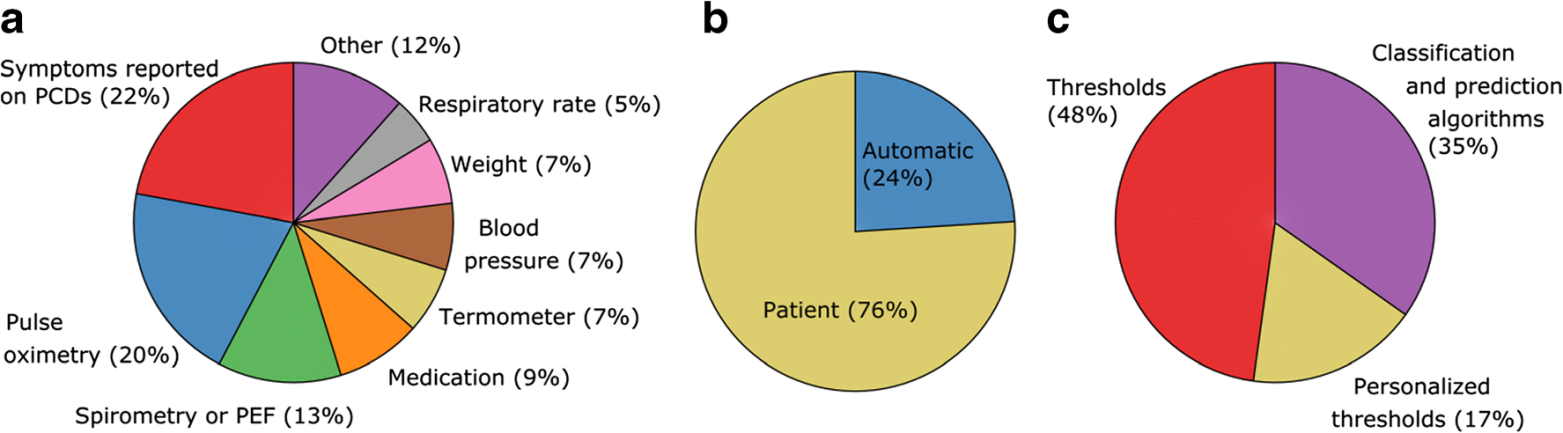
Overview of existing decision support systems. **a** Sensors used (Other: video/audio (five systems), physical activity (four systems), ECG (two systems), lung and heart sounds (one system), glucometer (one system)). **b** Patient action needed for data acquisition or automatic data acquisition. **c** Algorithms used. Total number of participant used in the studies is 1866, whereas the average duration of the studies was 6.4 months

In most of the existing remote-monitoring systems, the information obtained is analyzed by health caregivers. Only some of them provide automatic decision support systems [15], stand-alone or in combination with human analysis. Figure 6 presents the COPD-related decision support systems data obtained from two existing reviews [20, 21], and reports featuring decision support that came after the reviews [84, 103, 134–137]. The most often used inputs are the self-reporting of symptoms on a PCD, followed by pulse oximetry and spirometry (Fig. 6a). ECG has been used in only two publications: as a source of the features for exacerbation prediction [136], and to detect “clinical alert” (further details not provided) [138], whereas the PtcCO_2_ was not used at all.

Panel b shows that only about one quarter of the research featuring decision support provides automatic data acquisition. This is related to the frequency of data acquisition and analysis, which was almost exclusively on a daily basis, except in [139] where it was 3 h, in [140] where the interval could be varied depending on each patient’s needs, as well as in [136], which is the only report featuring continuous data processing. Patients’ compliance is affected by the frequency and method of data acquisition, as discussed in Sect. 4.3, but more importantly, it is not possible to detect immediate life-threatening events without continuous monitoring.

Clinical decisions are traditionally based on a set of predefined universal rules (see previous section). It is for that reason that the approach most often utilized for detecting and predicting exacerbation was by defining universal (population-based) thresholds on obtained symptoms and physiological parameters (panel c). These thresholds were sometimes adjusted to individual patients’ needs, but the best results were obtained by using more advanced classification algorithms: linear discriminant classification [141], a Bayesian network [140], a probabilistic neural network classifier [142], multilevel logistic regression [143], classification and regression trees [144], k-means clustering [145], a state machine combined with logistic regression [84], and a hybrid classifier combining a support vector machine, random forest, and a rule-based system [136]. The purpose is to classify the patient’s status as being in exacerbation (detection) or as transitional towards exacerbation, i.e., the prodromal period (prediction).

The reported accuracy in detecting exacerbations ranged from 40 to 94%, the sensitivity from 6 to 80%, and the specificity from 61 to 95%. The best accuracy in the early detection of exacerbations is reported for the hybrid classifier with 10 measured parameters and a total of 25 features used as the inputs [136]. Nevertheless, by using only self-reported symptoms as inputs, and k-means clustering, it is possible to obtain a sensitivity of 75% and 90% specificity for early exacerbation detection [145]. Furthermore, a pulse oximeter alone in combination with a classification algorithm can provide a high predictive accuracy [84].

Only two studies [142, 145] reported using patients’ electronic health records, but these were developed only for the purpose of the study and did not seem to be integrated into the patients’ standard care.

Besides completely outperforming the threshold approach and providing encouraging results, advanced classification algorithms ensure that the classification is adapted to each patient, provide ranking of features based on their predictive power [84], as well as the extraction of new rules [136]. For a concise description of the predictive analytics methods used in healthcare, the reader can refer to [21].

In addition to detecting exacerbations, it is beneficial to assess their severity for the purpose of deciding between home or hospital treatment.

### Assessment of severity and deciding between home or hospital treatment

The severity of an exacerbation is assessed crudely by tachypnoea, tachycardia, the use of accessory respiratory muscles, cyanosis, and evidence of respiratory muscle dysfunction or fatigue (e.g., uncoordinated ribcage motion or paradoxical movement of the abdominal wall during inspiration) [76]. If the severity of an exacerbation is in doubt, it should always be assessed in hospital. Referral to a hospital’s emergency department is mandatory in the case of respiratory failure indicated by the increased use of accessory respiratory muscles, paradoxical movement of the abdominal wall during inspiration, and significant deterioration in mental status.

Mild exacerbations of COPD are generally believed to represent an increase in symptoms, especially dyspnea, not necessarily accompanied by increased cough and sputum production, which might be more tenacious than usual. These parameters can be obtained through a self-assessment (see Sect. 4.5). Severe exacerbation, on the other hand, is associated with acute respiratory failure, especially in patients with an impaired lung function, sometimes accompanied by hypercapnia [7], which can be obtained from a PaCO_2_ measurement. Severe exacerbations cannot be treated at home, so if detected, patients should be transported to a hospital.

There are no definitive clinical guidelines about whether a patient should be cared for at home or in a hospital, and physicians are often uncertain when making this decision. The most important factors are the severity of the exacerbation, acute respiratory failure, the onset of new physical signs (e.g., cyanosis, peripheral edema), and the failure of an exacerbation to respond to initial medical management [30]. Other factors that can be taken into account are cause of the exacerbation (for example, severe pneumonia), a coexisting disorder that requires admission, degree of disability, social factors like the degree of support in the community (e.g., whether the patient lives alone), patient’s history, and mental state [7, 75].

### Detection of provoking and predictive factors

It is not clear which factors determine the development and severity of an exacerbation [7]:

- It is commonly thought that viral and bacterial infections of the tracheobronchial tree are the major causes of exacerbations in the later stages of disease [129]. The role of bacterial and viral infections in COPD exacerbations is still considered as controversial by some authors [7].
- Air pollution [66].
- There is some evidence that ozone concentration might be slightly associated with additional hospital admissions [146].
- Poor nutrition, i.e., malnutrition, in combination with respiratory muscle fatigue can aggravate the exacerbation.
- Drugs (especially tranquilizers).
- Stopping regular medication such as diuretics and/or bronchodilators on the patient’s own initiative can increase symptoms, which means that the monitoring of medication compliance is important (Sect. 4.6).
- Inappropriate oxygen administration can aggravate an exacerbation because of a reduction in the hypoxic respiratory drive.
- Conditions that can mimic or aggravate symptoms are pneumonia, pulmonary hypertension, heart failure or arrhythmias, pulmonary embolism, and pneumothorax.
- The cause of about one third of severe exacerbations of COPD cannot be identified [66].

A study involving 64 patients with moderate-to-severe COPD showed evidence that chronic hypercapnic respiratory insufficiency (high PaCO_2_) and pulmonary hypertension are predictive factors for hospitalization caused by COPD exacerbation [147]. Long-term oxygen therapy and perhaps even long-term noninvasive mechanical ventilation at home are possibly factors that reduce the risk of severe exacerbations, since there is evidence that they reduce hospital admission in COPD with chronic hypercapnia [148].

A study using the SGRQ showed that factors for predicting frequent exacerbations were daily cough, daily wheeze (clinical sign of exhaling difficulties caused by a narrowed tracheobronchial tree), and daily cough and sputum together, and frequent exacerbations in the previous year [149]. Another study showed that SGRQ scores and poor quality of life are associated with re-admission for COPD [150].

### Educational programs

Even though there are reports that educational programs for COPD patients can significantly reduce the utilization of healthcare services and improve health status [151], they have not been as actively promoted as much as programs for asthma patients [76]. Single-topic programs are available (e.g., smoking cessation, long-term oxygen therapy, rehabilitation), but there are insufficient integrated educational materials incorporating all the aspects of disease management [151].

Educational programs should improve people’s knowledge about the disease process and its treatment and should also motivate patients to change behavior and lifestyle, with the goal of improving their quality of life [151].

## Notes about normal and pathological ranges and changes in the measured physiological signals

### SaO_2_ and PaO_2_

The SaO_2_ normal range (two standard deviations (SDs) around mean) for adults aged < 70 years at sea level is 94–98%. For young adults (age 18–24), the 2SDs PaO_2_ range is 11.98–14.82 kPa (89.3–110.5 mmHg). The lower limit for this range decreases significantly with increasing age [152], e.g., the range for 64 years old and more is 9.02–14.76 kPa (67.3–110.1 mmHg) [47]. Additionally, the PaO_2_ is 0.8 kPa (6 mmHg) lower in the supine position than in the upright position.

Oxygen demand and oxygen delivery increase during exercise and reduce during rest and sleep. Hypoxemia (too low PaO_2_) can be defined as PaO_2_ below the normal lower limit, but most authors suggest values of less than 8 kPa (60 mmHg), or SaO_2_ of 90% [47].

The most common recommendation for patients with COPD is that oxygen is admitted if the resting awake PaO_2_ is less than 7.3 kPa (55 mmHg), or if it is 7.3–7.9 kPa (55–59 mmHg) in the presence of an elevated hematocrit (55%) or elevated right ventricular pressure evident from ECG [55].

### PaCO_2_

The reference range for PaCO_2_ is 4.6–6.1 kPa (34–46 mmHg) for a healthy adult of 18–38 years [47]. Values above 6.1 kPa are out of the normal range, but values up to 6.7 kPa can be accomplished by holding the breath.

### Body temperature

In humans, the traditional normal value for the oral temperature is around 37 °C [153]. Various parts of the body are at different temperatures, and the magnitude of the temperature difference between the parts varies with the environmental temperature [153]. There are a lot of other variables that can influence temperature measurements, such as the measurement site, time of the day, age, etc. [95].

A body temperature higher than 38.5 °C is one of the factors indicating severe exacerbations [76].

### Electrocardiogram

With the development of pulmonary heart disease due to COPD, the following changes might be seen in a routine ECG [154]:

- The P wave axis is farther right than + 75°.
- Any of the right ventricular hypertrophy criteria.
- Late R wave progression in precordial leads.
- Low voltage.
- Abnormal Q waves in the inferior or anterior leads.
- Supraventricular arrhythmias, especially atrial tachycardia, multifocal atrial tachycardia, and atrial fibrillation.
- Ventricular arrhythmias [155].

On the other hand, some more acutely presenting ECG changes may signal higher risk for the patient. It is known that patients with COPD have higher rates of cardiovascular diseases and consequently higher incidence of cardiovascular causes of mortality [63–66].

Some ECG changes can be attributable to acute overloading of the right ventricule of the heart which may be seen in acute pulmonary embolism or acute respiratory failure, such as

- S1Q3 pattern in standard leads.
- Acute right bundle branch block.
- Right axis deviation [155].

but some more subtle changes may contribute to the importance of the ECG changes in defining the risk of the patients with otherwise stable COPD:

- Prolongation or shortening of the heart rate corrected QT interval (QTc) suggests higher incidence of sudden cardiac death. It has been shown that these changes may occur more often in patients with COPD [156].
- Reduced heart rate variability is also a marker of sudden cardiac death and has been connected to patients with COPD.
- Dispersion of the QT interval in ECG recordings shows potential to foresee the adverse events and has been appreciated more often in patients with COPD [157].

Supraventricular arrhythmias occur frequently in patients with COPD [63]. Most often, they comprise atrial tachycardias, especially multifocal atrial tachycardia, atrial fibrillation, and atrial undulation which are often chronic. Ventricular tachycardias such as non-sustained ventricular tachycardia may depict patient’s higher risk for adverse events. Sustained ventricular tachycardia and ventricular fibrillation are arrhythmias that need to be terminated instantaneously. Arrhythmias are in general life-threatening events and can lead to dangerous complications. For instance, patients with COPD and multifocal atrial tachycardia have higher mortality rates [158].

Cardiac arrhythmia is a group of conditions in which the heartbeat is irregular, too fast, or too slow. If a heartbeat is above 100 beats/min in adults, it is called tachycardia and a heartbeat that is below 60 beats/min is called bradycardia. To distinguish between different arrhythmia types, other diagnostic procedures must be used in addition to the HR analysis.

The ECG can show signs of myocardial ischemia, specifically ST segment and T wave changes, as well as signs of myocardial infarction, specifically changes in the QRS pattern. However, not all patients with acute or previous myocardial infarction exhibit ECG changes [159, 160].

### Arterial blood pressure

“Hypertension is likely to be the most frequently occurring comorbidity in COPD and can have implications for prognosis” [30]. The pressure in the aorta and in the brachial and other large arteries in a young adult human rises to a peak value (systolic pressure) of about 120 mmHg during each heart cycle and falls to a minimum (diastolic pressure) of about 70 mmHg [161]. Conventional notation for the arterial pressure is systolic pressure over diastolic pressure, e.g. 120/70 mmHg. There are, however, a number of variables that can influence normal blood pressure values. For details, the reader is referred to [161] or similar literature.

There are different guidelines that usually define the pressure intervals specifying different hypertension severities. Stage 1 hypertension is defined as “clinical blood pressure of 140/90 mmHg or higher and subsequent ambulatory blood pressure monitoring daytime average or home blood pressure monitoring average blood pressure is 135/85 mmHg or higher” [162]. Therefore, in remote settings, we can consider 135/85 mmHg as a general threshold for hypertension.

### Spirometry output

The presence of a post-bronchodilator FEV_1_/FVC of less than 0.70 confirms the presence of COPD [66]. The severity of COPD can be categorized based on FEV_1_ (percent of predicted): mild ≥ 70, moderate 50–69, severe < 50 [76], or according to GOLD: mild ≥ 80, moderate 50–79, severe 30–48, very severe < 30. For the complete list of reference values in spirometry, see [91].

### Respiratory rate

One of the signs of COPD exacerbation is a respiratory rate over 25/min [76]. A normal adult human at rest breathes 12–15 times a minute [163].

## Conclusion and future development

The guideline for home treatment and management of mild COPD exacerbations, as specified by the European Respiratory Society [76], states that patients need to be reassessed every 48 h for worsening of symptoms, signs, and measurements. With the inclusion of continuous monitoring, this interval can be prolonged and the home treatment of exacerbations made more secure.

Two crucial improvements to the current remote-monitoring systems are enabling the continuous monitoring of the most important physiological parameters and enabling real-time decision support based on advanced classification algorithms, which are still to prove their clinical reliability. This will make it possible to detect life-threatening events in real time, consequently reducing mortality and hospitalization. Other expected consequences are an increase in the cost efficiency and an improvement of patients’ compliance with the monitoring. Furthermore, continuous monitoring can provide new insights into the patients’ states, like detecting transitions from a deteriorated to the normal state that happened without an intervention, and information about the durations of the exacerbations. Moreover, the decision support algorithms can rank features by their predictive power and even create new rules, consequently enriching clinical knowledge.

All the presented devices are already mature enough to be used for remote monitoring. Only in the case of transcutaneous measurements of O_2_ and CO_2_, the high costs of the devices currently hinder them from being placed in a large proportion of COPD patients’ homes. Still, as we discussed in Sect. 4.1, they could be efficiently and usefully applied by nurses visiting patients in their homes, and shared between multiple patients.

In the future, we can expect the design of the available devices to further improve in terms of lower power usage, the batteries will have higher capacities, enabling longer stand-alone periods for wireless sensors, and the electronics will improve, enabling further minimization. The level of user acceptance should be considered even during the design time, because tailoring the systems to target groups’ specific needs, reduces technophobia, and consequently improves acceptance.

At the signal-processing level, the obtained physiological signals are usually much more contaminated with noise and artifacts in the case of remote monitoring, with respect to those obtained in hospital settings. How to remove the artifacts and noise efficiently is still an open question, and algorithms are currently being developed for this purpose, which should run efficiently on sensors or PCDs. The possibility of having a lot of wireless sensors, either worn by the subjects or placed in subjects’ environments, that can produce the data quickly (e.g. ECG, 1000 samples per second) also imposes specific challenges in the area of wireless networks, which should be able to communicate the data without significant losses, and handle the devices in a plug-and-play manner.

The sensitive nature of medical data imposes a need to ensure the privacy and security of the obtained measurements at all levels, from sensors to the cloud, and in all communication channels. This is still an open issue for which algorithms, frameworks, and standards are currently being developed.

With the future developments of all the necessary technology, we can expect remote monitoring systems for COPD, as well as for other diseases, to become integrated into the healthcare system, which will reduce costs and improve care. Additionally, remote monitoring enables the treatments and care to be tailored to each patient’s needs based on their predicted response and individual risks, which is the core requirement of personalized medicine.
